# DNA Persistence and Relapses Questions on the Treatment Strategies of *Enterococcus* Infections of Prosthetic Valves

**DOI:** 10.1371/journal.pone.0053335

**Published:** 2012-12-31

**Authors:** Jean-Paul Casalta, Franck Thuny, Pierre-Edouard Fournier, Hubert Lepidi, Gilbert Habib, Dominique Grisoli, Didier Raoult

**Affiliations:** 1 URMITE, Aix Marseille Université, UMR CNRS 7278, IRD 198, INSERM 1095, Faculté de Médecine, Marseille, France; 2 Service de Cardiologie, Hôpital de la Timone, Marseille, France; 3 Service de Chirurgie Cardiaque, Hôpital de la Timone, Marseille, France; INSERM U1094, University of Limoges School of Medicine, France

## Abstract

We used amplification of the 16S rRNA gene followed by sequencing to evaluate the persistence of bacterial DNA in explanted heart valve tissue as part of the routine work of a clinical microbiology laboratory, and we analyzed the role of this persistence in the relapses observed in our center. We enrolled 286 patients treated for infective endocarditis (IE) who had valve replacement surgery and were diagnosed according to the modified Duke’s criteria described by Li et al. from a total of 579 IE cases treated in our center. The patients were grouped based on the infecting bacteria, and we considered the 4 most common bacterial genus associated with IE separately (144 were caused by *Streptococcus* spp., 52 by *Enterococcus* spp., 58 by *Staphylococcus aureus* and 32 by coagulase-negative *Staphylococcus)*. Based on our cohort, the risk of relapse in patients with enterococcal prosthetic valve infections treated with antibiotics alone was 11%. Bacterial DNA is cleared over time, but this might be a very slow process, especially with *Enterococcus* spp. Based on a comprehensive review of the literature performed on Medline, most reports still advise combined treatment with penicillin and an aminoglycoside for as long as 4–6 weeks, but there has been no consensus for the treatment of enterococcal infection of prostheses in IE patients.

## Introduction

Morbidity associated with infective endocarditis (IE) may extend beyond the successful treatment of active disease and hospital discharge, and it includes recurrence, heart failure, the need for valve surgery, and death in the long-term follow-up. Although many cases of IE can be cured with either medical therapies or combined medical and surgical therapy, patients who recover remain at risk for an additional episode of IE. The lifetime risk of a second episode of IE among survivors of IE has been estimated to be between 2% and 22% [1]. In different studies, recurrences were reported in 2.4% to 10.9% of the cases [1], [2], [3]. Repetitive IE due to the same species can represent relapse of the initial infection or de novo infection. Relapses are defined as repeat episodes of IE caused by the same microorganism responsible for the initial episode. Typically, in infectious diseases, the term “relapse” suggests an incompletely treated primary episode that results in the emergence of the original microorganism from a protected source. A diagnosis of relapsed IE suggests failed therapy and mandates a search for a persistent focus of infection (e.g., a valve ring abscess), a longer course of treatment, or surgical therapy. Bacterial culture of excised valvular material is a useful method for identifying the cause of IE and confirming the diagnosis from blood cultures. However, it is evident from the present study and others [4]–[8] that culture of valve tissue has only limited potential in the diagnosis of endocarditis, especially if antibiotic treatment is instituted before surgery. In our laboratory, we have diversified the tests used for the diagnosis of IE over the past few years [9]–[11] and have used broad-range PCR and sequencing to detect and identify bacterial DNA in the valves of patients treated for IE since 1994. In a previous study, performed between April 1, 1994 and April 31, 2003, we found that bacterial DNA persisted more frequently in patients who underwent valve replacement while on antibiotic treatment for IE (60%) than in patients who had completed antibiotic treatment for IE (37%; *p* = 0.02) [7]. The finding that bacterial DNA was more likely to be detected in the valves of patients with active IE than in patients who had completed antibiotic treatment for IE suggests that bacterial DNA is cleared slowly. In this report, we detail the results of a new examination of explanted heart valve tissue for streptococcal, enterococcal, *S. aureus–*associated and coagulase-negative staphylococcal (CNS) IE to evaluate the prevalence of DNA in cardiac valves. We performed a retrospective study on relapses to identify a possible link between DNA persistence and clinical relapse and performed an exhaustive review of the literature to develop a strategy to reduce relapses.

## Patients and Methods

### Patients

In this study, which includes cases from a previous study [7], we enrolled a total of 286 patients with endocarditis who underwent valve replacements on a series of 579 IE treated in our center. All patients were diagnosed according to the modified Duke’s criteria described by Li et al. [12]. We considered the 4 most common bacterial species associated with IE (*Streptococcus* spp., *Enterococcus* spp., *Staphylococcus aureus* and CNS) diagnosed by a microbiological endocarditis kit [10]. The patients had been or were being treated with antibiotics when they had valve replacement surgery. The antibiotic treatment for all of the patients was in accordance with our protocols [13], [14]. Days of treatment with parenteral antimicrobials with more than one active antibiotic were counted. A follow-up of these 579 patients was organized for one year (Table 1).

**Table 1:** Patients with IE seen at our center.

	No surgery	Prosthetic valve not removed	Valve removed	Prosthetic valve removed	Total
***Streptococcus*** ** spp.**	**139**	64	**144**	31	**283**
***Enterococcus*** ** spp.**	**39**	18	**52**	17	**91**
***S. aureus***	**71**	49	**58**	19	**129**
**CNS**	**44**	28	**32**	24	**76**
	**293**	159	**286**	91	**579**

### Microbiology

Three aerobic (BACTEC PLUS Aerobic/F) and anaerobic (BACTEC LYTIC/10 Anaerobic/F) blood cultures were obtained from each patient and incubated for 1 week in a BACTEC 9240 Blood Culture System (Becton Dickinson, Sparks, MD, USA). Valve tissue from patients was inoculated onto 5% blood agar (bioMerieux, Marcy-l’Etoile, France) and chocolate agar (bioMerieux) and incubated at 37°C in a 5% CO_2_ atmosphere for 10 days. Valve samples were also cultured on BCYE (bioMerieux) for 15 days and on Columbia media for 10 days under anaerobic conditions.

### PCR Amplification and Sequencing

DNA was extracted from the heart valve samples using a QIAmp Kit (QIAGEN, Hilden, Germany) or a FastDNA kit (Bio 101, Carlsbad, CA). DNA was amplified using *Taq* DNA polymerase (Gibco BRL, Life Technologies) and broad-range primers specific for the 16S rRNA gene 536f (5′-CAG CAG CCG CGG TAA TAC-3′) and 1050r (5′-CAC GAG CTG ACG ACA-3′). The sequences obtained from the amplified DNA were compared with those available in GenBank using BLASTn (http://blast.ncbi.nlm.nih.gov/). PCR was also conducted on negative controls (DNA isolated from valves of patients without IE), usually with one control for every 5 patient samples. Positive amplification of any negative control caused the experiment to be considered unreliable, and only DNA corresponding to the bacterium responsible for the patient’s IE was considered a positive result.

### Histopathology

One histopathologist examined all hematoxylin/eosin-stained sections of the valves and classified lesions as (i) consistent with IE, (ii) having no signs of IE, or (iii) indeterminate according to criteria defined elsewhere [15]. Specific stains, such as Gram and Whartin-Starry, were used when necessary [15].

### Statistical Analysis

Demographic and clinical data, etiological agents (blood and valve culture results and serology), and duration of antibiotic treatments were compared using the chi-squared or Mann-Whitney tests according to PCR results. Univariate and multivariate logistic regressions were used to assess the situations more likely to benefit from systematic PCR amplification of the 16S rRNA gene and sequencing of the valves. The variables evaluated were age, sex, duration of antibiotic treatment, positive blood cultures, and histology. STATA software (version 7.0) was used for analysis.

### Relapse

Relapse was defined as a new episode of endocarditis caused by the same bacteria observed in the initial case after completion of treatment, as detected based on the blood or valve cultures.

### Bibliography

An exhaustive bibliography was assembled using Medline based on our preliminary results. The keywords used were “relapse,” “*Enterococcus*,” “prosthetic valve,” and “endocarditis.” After reading and analyzing the abstracts, we sorted the reports into a large series detailing incidences of relapse, and we selected case reports reporting enterococcal IE.

### Ethic Statement

The study was approved by the Ethic Committee of the Institute Fédératif de Recherche 48, Marseilles, France (Agreement #07–015).

Verbal consent is required for patients. These patients arrive in the emergency and cardiology department and are not able to sign any consent whatsoever. The ethics committee approved this procedure consents.

## Results

A total of 286 patients who underwent valve replacement were studied from a series of 579 IE cases. The blood cultures were positive for 286 patients, including 144 with *Streptococcus* spp., 52 with *Enterococcus* spp. (47 *E. faecalis*, 3 *E. faecium*, and 2 *E. durans*), 58 with *S. aureus* and 32 with CNS (Table 1). All patients had been or were being treated with antibiotics when they had the valve replacement surgery. Bacterial DNA was amplified in 204/286 patients (70.2%) (Table 2). To determine the effect of current antibiotic treatment on the PCR results, we analyzed the data on 245 patients with IE who were receiving antibiotic treatment at the time of surgery and the data on the 41 patients who had completed antibiotic treatment at the time of surgery. The duration of antibiotic treatment for all patients was 45 days, in accordance with the protocols used at our center [13]. Bacterial DNA was amplified from 124/153 (81%) patients receiving antibiotics for ≤15 days, from 61/92 (66.3%) patients receiving antibiotics for >15 days but who had not yet completed antibiotic treatment and in 16/41 (39%) patients who had completed antibiotic treatment. We found that patients with ≤15 days antibiotic therapy, ≥15 days but not completed therapy and completed treatment have significantly decreasing positive PCR results (*p*<0.00001) (Table 2). In the 245 patients with IE who were receiving antibiotic treatment at the time of surgery, *Streptococcus* spp. and *Enterococcus* spp. accounted for 117 and 49 cases, respectively, and *Staphylococcus aureus* and CNS accounted for 48 and 31 cases, respectively. Table 2 shows the PCR results the patients grouped based on the infecting bacteria and the delay between the beginning of antibiotic treatment and surgery. The valves of patients infected with *Streptococcus* spp. (97/117; 82.9%), *Enterococcus* spp. (36/49; 73.4%) and *Staphylococcus aureus* (35/48; 72.9%) were more likely to be PCR positive than those infected with CNS (17/31; 54.8%; *p*<0.00001). Of the 41 patients who had completed antibiotic treatment at the time of surgery, 16 presented with positive PCR tests. Twelve of these sixteen patients were infected with *Streptococcus* spp., three with *Enterococcus* spp., and one with *Staphylococcus aureus*; none were infected with CNS (Table 2). Patients with enterococcal IE were significantly more likely to be PCR positive (3/3) than patients with IE caused by other organisms (13/38; *P*<0.03). Table 2 shows the results of cultures performed on heart valves from 286 patients; bacterial DNA could be amplified from 32 cases (11.2%). Of the 153 patients who had been on antibiotics for less than 15 days prior to surgery, bacterial DNA could be amplified from 26 (17%). Bacterial DNA could be amplified from 4 of the 92 patients (4.3%) who had been on antibiotics for at least 15 days prior to surgery but had not yet completed the antibiotic regimen and 2 of the 41 (7.3%) patients who had completed the antibiotic course prior to surgery. A case with a positive culture for CNS but negative PCR results did not show histopathological evidence of endocarditis; therefore, it was considered a result of bacterial contamination. Cases of culture-positive *Enterococcus* spp. were considered a failure of antibiotic therapy, and there were significantly more cases of relapse associated with *Enterococcus* spp. after completed therapy (1/3) compared with other organisms (0/38; *P*<0.001).

**Table 2:** Comparison of the PCR and valve bacteriologic culture results performed on heart valves from 286 patients at the time of surgery.

Positive PCR (%) by species	≤15 days	Incomplete treatment	Completed treatment	*p*
***Streptococcus*** ** spp. 144**	60/69 (87%)	37/48 (77%)	12/27 (44%)	0.00007
***Enterococcus*** ** spp. 52**	27/33 (82%)	9/16 (56%)	3/3 (100%)	0.1
***S. aureus*** ** 58**	25/32 (78%)	10/16 (62%)	1/10 (10%)	0.0005
**CNS 32**	12/19 (63%)	5/12 (41%)	0/1 (0%)	0.3
**Total: 201/286 (70.2%)**	**124/153 (81%)**	**61/92 (66.3%)**	**16/41 (39%)**	**<0.00001**
**Cultures of valves**				
***Streptococcus*** ** spp.**	6/69 (8%)	2/48 (4%)	0/27 (0%)	
***Enterococcus*** ** spp.**	5/33 (15%)	0/16 (0%)	1/3 (33%)	
***S. aureus***	5/32 (15%)	1/16 (6%)	0/10 (0%)	
**CNS**	10/19 (52%)	1/12 (8%)	1/1	
**Total: 32/286 (11.2%)**	**26/153(17%)**	**4/92 (4.3%)**	**2/41(7.3%)**	**0.002**

However, there was a significant decrease in the number of positive cultures as the time between the initial treatment of IE and the valve surgery increased *(P* = 0.002). During the follow up of the 579 patients, we identified four cases of relapse in patients who had completed antibiotic treatment but not undergone cardiac surgery during their initial case of IE; these relapses were caused by *Streptococcus viridans* and *Enterococcus faecalis* (Table 3). We did not observe any relapse in the patients who benefited from valve replacement surgery during the initial occurrence of IE. The four observed relapses were among 178 patients with *Streptococcus viridans*– or *Enterococcus faecalis–*associated IE who received complete medical treatment but no cardiac surgery after the initial presentation of IE. Two of these cases were *Streptococcus* spp. relapses (2/139; 1.4%), one of which was an infected prosthetic valve, and two cases were *Enterococcus faecalis* relapses (2/39; 5.1%), both of which were infected prosthetic valves (Table 1). We report here only the two cases of enterococcal IE relapse.

**Table 3:** Relapses after medical treatment.

Cases	Bacteria	Prosthetic valve	PCR	Valve culture	Anatomopathology	Date of relapse
**1**	*S. anginosus*	No	+	–	+	60 days
**2**	*S. oralis*	Yes	+	–	+	51 days
**3**	*E. faecalis*	Yes	+	–	–	27 months
**4**	*E. faecalis*	Yes	+	+	+	57 days

### Case Reports

A 76-year-old man presented with *E. faecalis*–associated prosthetic aortic valve endocarditis. He was treated with ampicillin and gentamicin for 45 days. Twelve days after the end of the treatment, *E. faecalis* reappeared in the blood cultures. He received another 45 days of combined treatment, after which the prosthetic aortic valve was replaced. *Enterococcus faecalis* DNA was amplified from the removed valve.

A 72-year-old woman was treated for *E. faecalis–*associated prosthetic aortic valve endocarditis. She was treated with ampicillin and gentamicin for 15 days then ampicillin alone for 30 days. Clinical symptoms of relapse started 27 months after treatment, and *E. faecalis* reappeared in the blood cultures. Five months after the prosthetic aortic valve was replaced due to cardiac insufficiency, she was treated with ampicillin and gentamicin for 45 days. *E. faecalis* DNA could be amplified from the valve. These 2 cases detailing patients with enterococcal IE suggest that the presence of a prosthetic valve is a higher risk factor of relapse than the failure to complete synergistic aminoglycoside treatment (4–6 weeks).

A comprehensive bibliography was assembled using Medline. Using the search phrase “relapse and endocarditis,” we obtained 1,030 articles. After critically reading the abstracts, we selected and analyzed the full text of 9 articles and compiled the results of large studies with relapse, case reports with relapses, as well as the bacteria responsible for the relapses (Table 4). While few case studies disclosed relapses, the reported incidences were 1.1% [16], 2.7% [17] and 3.3% of patients [3] (Table 4). In another study, the authors [18] reported two cases of enterococcal IE relapse in native valves. The authors concluded that the treatment of similar cases of endocarditis should be extended beyond 6 weeks to prevent any recurrence of the disease. Using the search phrase “relapse, prosthetic valve, endocarditis,” we identified 243 articles. We analyzed the abstracts to determine whether prosthetic valves could be a risk factor for relapse in patients with IE. The relapse rate in prosthetic valve endocarditis may be higher than in native valve endocarditis [3]. Next, to determine the number of patients with prosthetic valve enterococcal IE relapses reported in the literature, we used the search phrase “relapse, Enterococcus, prosthetic valve, endocarditis”; a total of ten case reports [19] and two larger studies were obtained. In 2007, a study [20] aimed to determine the risk factors for mortality in patients with enterococcal endocarditis. The authors reviewed 47 cases of enterococcal endocarditis in 44 patients from a retrospective cohort study. They compared cases of native valve and prosthetic valve endocarditis and found no differences regarding complications, the need for surgical treatment, or mortality; 8 of the 44 patients (18%) died. In this study, the authors do not consider prosthetic valves to be a risk factor for relapse in patients with enterococcal IE. Another study conducted in Sweden between 1995 and 1999 [21] reported 93 cases of enterococcal endocarditis. The incidence of mortality during treatment was 16%, and the relapse rate was 3% (3 cases). We report the description of these 3 case reports taking from the literature to exemplify the study by the real case of relapses.

**Table 4:** Relapses reported in the literature.

Ref	Number of patients	Type ofstudy	Number of relapses	Relapse*S. aureus*	Relapse*Streptococcus* spp.	RelapseNCS	Relapseother bacteria	RelapseProsthetic valve	Relapse*Enterococcus* spp.	Relapse*Enterococcus* spp.Prosthetic valve
**Mansur**1978–1999	420	serie	14	3	4	3	1	7	3	NR
**Chu**1986–2004	428	serie	9	6	0	0	1	0	2	0
**Pilar Tornos**1975–1990	112	serie	3	1	1	0	1	0	0	0
**Ho**2000–2007	80	serie	5	5	0	0	0	5	0	0
**Casalta**1994–2010	579	serie	4	0	2	0	0	3	2	2
**Fernandez**	47	Enterococcal IE serie	3	0	0	0	0	NR	3	NR
**Lars Olaison**1995–1999	93	Enterococcal IE serie	3	0	0	0	0	1	3	1
**JP Talarminin**2011	1	Case report	1	0	0	0	0	1	1	1
**Jovanovic**2010	2	Case report	2	0	0	0	0	0	2	0
**Total**	**1762**	**X**	**44 (2.49%)**	**15** **(34%)**	**7** **(16%)**	**3** **(6.8%)**	**3** **(6.8%)**	**17** **(38%)**	**16** **(36%)**	**4** **(25%)**

NR: Non Referred.

A 41-year-old woman was treated for *S. aureus*–associated endocarditis with spondylitis; 39 months later, she presented with *E. faecalis–*associated native aortic valve IE with a time to treatment of 15 days. She developed heart failure and had a prosthetic aortic valve implanted on day 5. She was treated with ampicillin and an aminoglycoside for 34 days. However, 22 days after treatment, *E. faecalis* reappeared in the blood cultures, and she received another 34 days of combined treatment. A replacement of the prosthetic aortic valve was performed 15 days after treatment. She died as a result of a cerebral embolism 13 days after this operation.

An 87-year-old man was treated for *Streptococcus mutans*–associated endocarditis; 31 months later, he presented with *E. faecalis*–associated native mitral valve IE with only 3 days of clinical symptoms. He was treated with 28 days of ampicillin, and an aminoglycoside was provided for only 7 days. The clinical symptoms of relapse started 6 days after treatment. He was then treated with ampicillin and an aminoglycoside for 28 days, followed by teicoplanin for 14 days. He recovered, but lifelong amoxicillin prophylaxis is planned. In this patient, clinical failure with relapse is probably the result of the short duration of the combined aminoglycoside therapy.

The third relapse was that of a 79-year-old man with early *E. faecium*–associated prosthetic aortic valve endocarditis whose treatment was delayed 60 days. He was treated with vancomycin for 42 days; this was combined with an aminoglycoside for 27 days. However, a relapse was diagnosed 69 days after treatment, with *E. faecium* detected in blood cultures; the patient died. The authors concluded that in patients with complicating factors, such as prosthetic valves, large and dense vegetation after long treatment delays, or decreased sensitivity to available antibiotics, a synergistic aminoglycoside treatment regimen might still be given. For enterococcal IE, most studies still advise combined treatment with amoxicillin and an aminoglycoside for as long as 4–6 weeks.

## Discussion

*Enterococcus* spp. are one of most common bacterial causes of IE. At our center, we have had 91 cases of enterococcal IE and a total of 579 cases of IE (Table 1), and *Enterococcus* spp. were the third leading cause of IE (15.7% of cases) (Table 1). In both our patient population and in the literature [20], [21], enterococcal IE has a high rate of mortality (15%–18%) and incidence of relapse (Table 4). Because the progression of enterococcal IE is slow, some patients have symptoms for a prolonged time before they seek medical care. The relapse rate may be higher in patients who have had symptoms of endocarditis for more than 3 months before treatment. [22]. Patients with enterococcal IE were more likely to have a positive PCR test and bacterial DNA that persisted for longer than patients with IE caused by other species (e.g., *Staphylococcus aureus* and CNS). Furthermore, in patients who had completed the treatment regimen, the PCR tests were positive more frequently in patients with enterococcal IE compared with patients with IE caused by other species (*p* = 0.03). A recent study [5] has confirmed this long-term persistence, particularly in IE cases caused by *Enterococcus* spp. The relapses raise the question of the persistence of DNA. In our review of the literature, we identified 44 reported relapses (Table 4); 16 of which (36%) were caused by *Enterococcus* spp. Relapses of prosthetic valve enterococcal IE were observed in 4/16 patients (25%) (Table 4). The relapse rate in prosthetic valve endocarditis may be higher than in native valve endocarditis [2] (Table 4). In our study, relapses of *E. faecalis*–associated IE were observed 2/18 (11%) patients with prosthetic valves with who did not have surgery at their initial presentation (Table 4). We did not observe any relapses in patients in whom valve replacement was performed during the initial treatment of IE. In our experience, unlike in other reports [23], patients with *Staphylococcus aureus*–associated prosthetic valve IE who received surgical treatment for the initial episode of endocarditis had a significantly reduced probability of relapse after following our treatment recommendations. These relapses suggest failed therapy. The risk of relapse is significantly higher in *Enterococcus faecalis*–associated prosthetic valve IE, and *E. faecalis* DNA could be detected in cardiac valves even after the patients completed antibiotic treatment (Table 2). The risk to have two successive IE on prosthetic valves in the year is very small. If we consider that, the risk is 1% for each episode and we have 15% of Enterococcus endocarditis in our series. The risk is estimated approximately to 10–7 (1%/yearX1%X 15%). The relapses are probably correlated with long-term persistence of DNA [3] but the relapses correlate with long-term persistence of DNA are difficult because we observed 2 relapses among the 3 patients, what seems to be informative even if we do not have the possibility to perform a statistical analysis, the number of samples were carried out with a small number. In the literature, a large set of nucleic acid detection methods with good sensitivity and specificity that are now available for the detection of pathogens. Many efforts have been made to combine these methods to assess viability. Genomic DNA PCR amplification has been shown to be inappropriate for distinguishing viable from dead bacteria owing to DNA stability. Many authors have tried to bypass this difficulty by switching to RNA amplification methods such as reverse transcription-PCR and nucleic acid sequence-based amplification [24]. Both antibiotics and valve surgery are used in the treatment of prosthetic valve IE [25]–[27], but it is still unclear if prosthetic valve enterococcal IE can be treated with antibiotics alone. It appears that combination therapy is optimal for the treatment of enterococcal IE [22]. A combination of cell wall–targeting agents (e.g., penicillin, ampicillin, or vancomycin) and aminoglycosides has been the standard for treatment of enterococcal IE since the first demonstration of the penicillin/streptomycin synergy in 1947 [28]. Although there has never been a controlled trial of combination therapy versus monotherapy in the treatment of enterococcal IE, it is generally believed that combination therapy is necessary for the treatment enterococcal IE because enterococci are tolerant to penicillins and glycopeptides [22]. In support of this view, high relapse rates (30% to 60%) have been reported for patients with enterococcal IE treated with amoxicillin alone [29].

Most recommendations still advise combination treatment with amoxicillin and an aminoglycoside for as long as 4–6 weeks [13], [30]. However, there is no consensus regarding surgical treatment of prosthetic valve enterococcal IE. The risk of death is between 15% and 18% [31]. In our population, the mortality of prosthetic valve enterococcal IE was 16.6%, and the relapse rate was 11% in patients treated with antibiotics alone. In one relapse in our patient population, clinical failure was probably the result of the short duration of combined aminoglycoside therapy. It appears that the presence of a prosthetic valve, decreased sensitivity to available antibiotics, and failure to complete a synergistic aminoglycoside treatment (4–6 weeks) are risk factors for relapse. Surgery without active infection could be a potential treatment of prosthetic valve enterococcal IE because in these cases the mortality rate is identical of the operative mortality after a second intervention for prosthetic dysfunction [32], [33]. The operative mortality rate evaluated by the EuroSCORE [34] is high, which probably precludes proposing surgery for the purpose of avoiding relapse. Antibiotic treatment with ampicillin for one year may be proposed for these patients to prevent relapse, but the effectiveness of this treatment remains to be evaluated because we have no reference in the literature. One year, because this treatment by amoxicillin is reported in the literature in cases of rheumatic fever and splenectomized patients.

### Conclusions

A diagnosis of relapsed IE suggests failed therapy and mandates a search for a persistent focus of infection (e.g., a valve ring abscess), a longer course of treatment, or surgical therapy. Our results show that as the time between the successful treatment of IE and valve surgery increases, the likelihood of bacterial DNA being detected in heart valves decreases. This indicates that bacterial DNA is cleared over time but that this might be a very slow process, especially in prosthetic-valve enterococcal IE. The results of our study and our review of the literature detailing patients with enterococcal IE suggest that the presence of a prosthetic valve, decreased sensitivity to available antibiotics, and failure to complete synergistic aminoglycoside treatment (4–6 weeks) are risk factors for relapse. The benefits of treatment with either cardiac surgery or ampicillin for one year may be discussed. We propose as an alternative therapeutic in patients with a high operative risk of mortality, to prescribe amoxicillin orally during 1 year.
